# Integrins Modulate T Cell Receptor Signaling by Constraining Actin Flow at the Immunological Synapse

**DOI:** 10.3389/fimmu.2018.00025

**Published:** 2018-01-18

**Authors:** Katarzyna I. Jankowska, Edward K. Williamson, Nathan H. Roy, Daniel Blumenthal, Vidhi Chandra, Tobias Baumgart, Janis K. Burkhardt

**Affiliations:** ^1^Department of Pathology and Laboratory Medicine, Children’s Hospital of Philadelphia and Perelman School of Medicine at the University of Pennsylvania, Philadelphia, PA, United States; ^2^Department of Chemistry, University of Pennsylvania, Philadelphia, PA, United States

**Keywords:** integrin, actin, immunological synapse, T cell, signaling, talin, vinculin, costimulation

## Abstract

Full T cell activation requires coordination of signals from multiple receptor–ligand pairs that interact in parallel at a specialized cell–cell contact site termed the immunological synapse (IS). Signaling at the IS is intimately associated with actin dynamics; T cell receptor (TCR) engagement induces centripetal flow of the T cell actin network, which in turn enhances the function of ligand-bound integrins by promoting conformational change. Here, we have investigated the effects of integrin engagement on actin flow, and on associated signaling events downstream of the TCR. We show that integrin engagement significantly decelerates centripetal flow of the actin network. In primary CD4^+^ T cells, engagement of either LFA-1 or VLA-4 by their respective ligands ICAM-1 and VCAM-1 slows actin flow. Slowing is greatest when T cells interact with low mobility integrin ligands, supporting a predominately drag-based mechanism. Using integrin ligands presented on patterned surfaces, we demonstrate that the effects of localized integrin engagement are distributed across the actin network, and that focal adhesion proteins, such as talin, vinculin, and paxillin, are recruited to sites of integrin engagement. Further analysis shows that talin and vinculin are interdependent upon one another for recruitment, and that ongoing actin flow is required. Suppression of vinculin or talin partially relieves integrin-dependent slowing of actin flow, indicating that these proteins serve as molecular clutches that couple engaged integrins to the dynamic actin network. Finally, we found that integrin-dependent slowing of actin flow is associated with reduction in tyrosine phosphorylation downstream of the TCR, and that this modulation of TCR signaling depends on expression of talin and vinculin. More generally, we found that integrin-dependent effects on actin retrograde flow were strongly correlated with effects on TCR signaling. Taken together, these studies support a model in which ligand-bound integrins engage the actin cytoskeletal network *via* talin and vinculin, and tune TCR signaling events by modulating actin dynamics at the IS.

## Introduction

T cell activation involves interaction of the T cell receptor (TCR) with peptide–MHC complexes displayed on the surface of an antigen-presenting cell (APC). This interaction takes place at a specialized cell–cell contact site termed the immunological synapse (IS), which serves as a platform for signaling events leading to T cell proliferation, differentiation, and effector function. Importantly, engagement of the TCR by peptide–MHC complexes is not sufficient to induce full activation of naïve T cells; additional costimulatory signals are needed. These costimulatory signals lower the dose of antigen required for full T cell activation and tune the functional outcome of activated cells (1). Proper signaling through the TCR and costimulatory receptors also requires the action of integrins, which interact with receptors on the APC to provide the sustained cell–cell contact needed for full T cell activation. Thus, multiple receptor–ligand interactions take place in parallel, within dynamic subdomains of the IS. Although it is clear that crosstalk among these molecules is essential for proper T cell activation, the mechanisms through which this occurs remain poorly understood.

T cells express two major integrins: LFA-1 (αLβ2, CD11a/CD18) binds to ICAM family proteins, while VLA-4 (α4β1, CD49d/CD29) binds to several ligands, including VCAM-1. Binding of LFA-1 to ICAM-1 is the predominant receptor–ligand interaction responsible for sustained T cell–APC adhesion (2). In addition to promoting cell–cell adhesion, engagement of LFA-1 by ICAM-1 delivers specific intracellular signals (the so-called “outside-in signals”) that lower the threshold dose of antigen needed to achieve full T cell activation, and influence T cell fate (3–9). In this respect, the adhesion molecule LFA-1 also serves as a costimulatory molecule. VLA-4 is expressed at appreciable levels only after T cell activation, and plays a crucial role in T cell trafficking (10). Nonetheless, there are reports that VLA-4 can also deliver costimulatory signals (11–13).

While it is clear that integrin-dependent adhesion and signaling modulates the outcome of TCR engagement (10), the mechanisms that mediate functional linkage between integrins and the TCR are incompletely understood. Many studies addressing this question have focused on signaling molecules such as SLP-76 and ADAP that interact with both receptors (7, 8, 14, 15). However, both receptors also interact with the actin cytoskeleton. TCR engagement induces actin polymerization, which in turn drives spreading of the T cell on the APC surface, organizes the assembly of signaling complexes, and coordinates the dynamic architecture of the IS (16–18). The β-chains of LFA-1 and VLA-4 bind tightly to actin filaments through the action of proteins, such as talin and vinculin (19). In addition, LFA-1 engagement induces formin-dependent actin polymerization and induces the formation of local “actin clouds” (7, 8, 20). Recent studies have shown that actin polymerization at the IS exerts pushing and pulling forces that are associated with receptor activation (21–25). This raises the possibility that the actin cytoskeleton serves as a mechanical intermediate in receptor crosstalk.

There are multiple distinct pools of dynamic actin filaments at the IS (26, 27). The most prominent of these is the lamellipodial actin network assembled by WAVE2-dependent Arp 2/3 complex activity (28). This network is assembled *via* the addition of actin monomers onto branched filaments near the edge of the spreading T cell. The network flows centripetally, driven by actin polymerization and organized by myosin II-based contractility (29, 30). This process, in turn, drives the movement of signaling complexes toward the central region of the synapse, where TCR signaling is extinguished and vesicular trafficking takes place (31). As part of this process, LFA-1 associates with branched actin filaments and formin-nucleated acto-myosin arcs at the lamellipodial edge, and moves inward to form an adhesive ring that surrounds the central region where TCR accumulates (23, 27). Unlike the TCR, which interacts weakly with F-actin and appears to be swept inward indirectly, activated LFA-1 molecules are physically linked to the dynamic F-actin network (32).

The movement of proteins at the IS is driven by the T cell actin cytoskeleton, which exerts radial forces in the pN–low nN range (33–35). In addition to exerting force parallel to the plane of the IS, actin drives pushing and pulling forces perpendicular to the IS (21, 24–26, 36). Increasingly, it is becoming clear that these mechanical forces are essential for T cell activation. Though the details are debated, there is evidence that force induces TCR deformation and/or exposes ITAMs within the CD3 complex [reviewed in Ref. (18)]. In addition, force has been proposed to foster serial triggering by enhancing TCR clustering and selectively promoting interactions with agonist peptides (22, 37). Integrin responses also involve an important mechanical component. We recently showed that centripetal flow of the T cell actin network exerts force on LFA-1, promoting the conformational changes needed for high-affinity ligand binding and costimulatory signaling, and organizing its spatial arrangement at the IS (23). Furthermore, we showed that this process is augmented by the APC cytoskeleton, which restricts the movement of ICAM-1, thereby providing a stationary counterforce that increases tension on LFA-1 and promotes its activation (38).

Since actin flow activates integrins by pulling on their cytoplasmic tails, we reasoned that the converse should also be true—integrin engagement should exert drag on the flowing actin network. In support of this idea, Nguyen et al. showed that co-engagement of TCR and VLA-4 dramatically slows centripetal flow of the actin network in Jurkat T cells (39). Although LFA-1 is the integrin primarily responsible for adhesion and costimulation at the IS, the effect of LFA-1 engagement on actin flow at the IS has not been carefully addressed. Moreover, the effect of integrin ligand mobility on IS actin dynamics has not been explored. Here, we use a combination of stimulatory surfaces to probe the contribution of β1 and β2 integrin engagement on actin flow and TCR signaling. We show that in primary T cells, engagement of either LFA-1 or VLA-4 significantly slows actin flow, especially when integrin ligands are immobile. This process involves mechanical coupling of integrins to the actin network *via* talin and vinculin. Finally, our studies reveal a direct correlation between actin flow rates and TCR-dependent signaling events. Taken together, our results provide support for a model in which the dynamic actin cytoskeleton serves as a mechanical intermediate for crosstalk between integrins and the TCR.

## Results

### Integrin Engagement Slows Actin Flow at the IS

**Figure 1:**
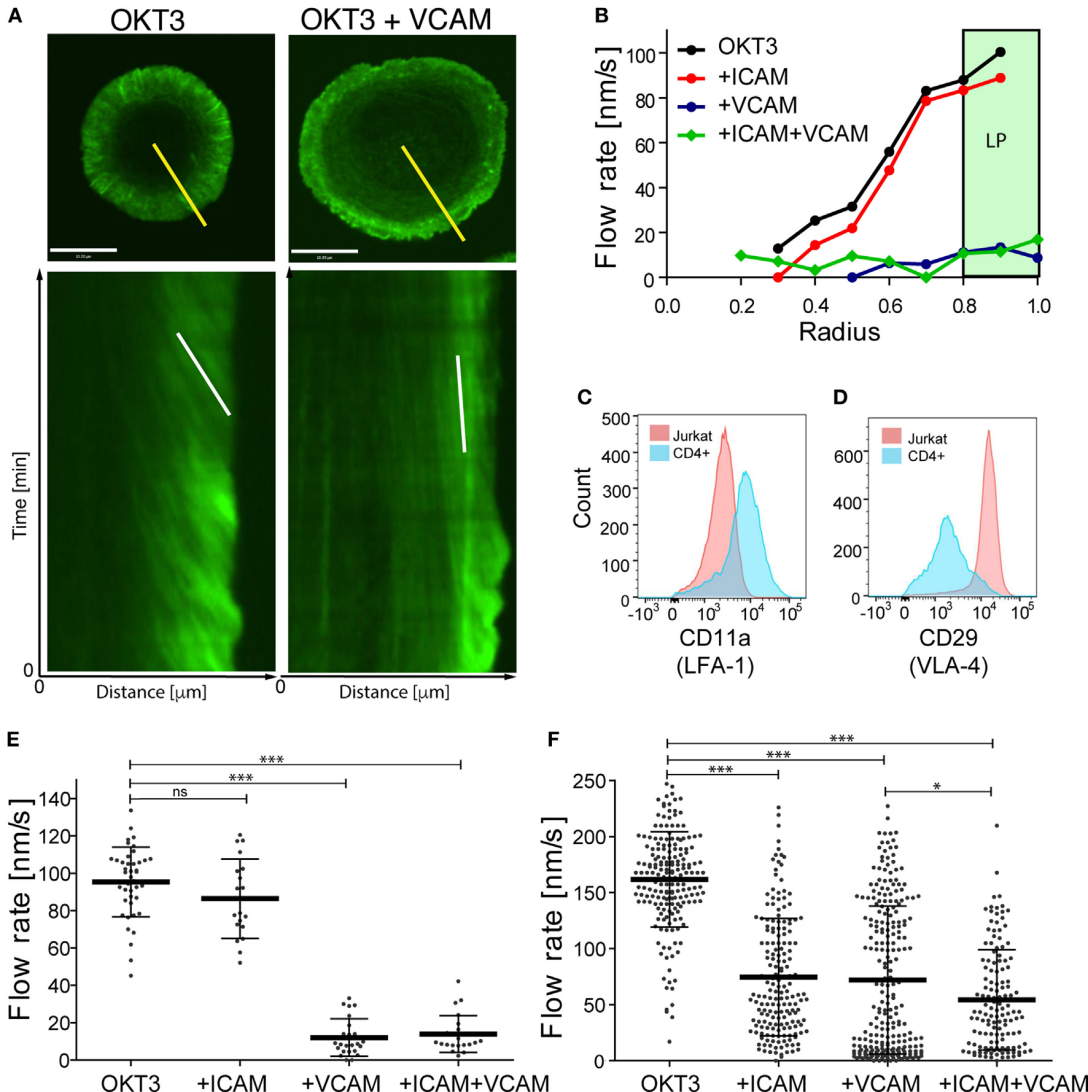
Integrin engagement slows actin flow. Jurkat T lymphoma cells and primary human CD4^+^ T cell blasts were allowed to interact with coverslips coated with anti-CD3 (OKT3) plus ICAM-1, VCAM-1, or both, and imaged for 4 min. **(A)** Still images of responding Jurkat cells stimulated on coverslips coated with anti-CD3 alone (left) or in the presence of VCAM-1 (right), together with corresponding kymographs of F-actin dynamics generated along the yellow lines. The white lines in the kymographs show example slopes used to calculate actin flow rates. **(B)** Kymographic analysis of F-actin features in Jurkat cells, showing the distribution of F-actin velocity across the immunological synapse (IS). The marked area displays the peripheral lamellipodial region (LP). **(C,D)** Surface expression of integrin LFA-1, as detected by CD11a staining and VLA-4, as detected by CD29 staining on Jurkat T cells (pink) and primary human CD4^+^ T cell blasts (blue). **(E,F)** Actin flow rates in the LP region for Jurkat cells **(E)** and primary human CD4^+^ T cell blasts **(F)**. Error bars show mean ± SD. *p < 0.05, ***p < 0.001. Scale bar = 10 µm.

We showed previously that actin flow at the IS influences integrin activation *via* a mechanical coupling mechanism (23, 38). We, therefore, wondered whether the converse is also true—i.e., whether integrin engagement influences actin flow, and if so, whether the β1 and β2 integrins VLA-4 and LFA-1 behave similarly. To test this, we conducted careful side-by-side analysis of T cells stimulated with VCAM-1 (a ligand for VLA-4) and ICAM-1 (a ligand for LFA-1). Jurkat T cells stably expressing GFP–actin were imaged by spinning disk confocal microscopy while spreading on glass coverslips coated with anti-CD3 alone, or together with either ICAM-1 or VCAM-1. Actin flow rates were determined as a function of cell radius based on kymographic analysis, as described in Section “Materials and Methods” and detailed in Ref. (40). As we reported previously (30), Jurkat cells spreading on anti-CD3 alone exhibited fastest actin flow in the peripheral lamellipodial region; flow rates decreased with inward movement until a central region of low actin density was reached (Figures 1A,B; Video S1 in Supplementary Material). Addition of VCAM-1 to the anti-CD3 coated stimulatory surface dramatically slowed actin flow across the entire IS. By contrast, addition of ICAM-1 did not slow actin flow; flow rates were indistinguishable from cells stimulated with anti-CD3 alone (Figure 1B; Videos S2 and S3 in Supplementary Material). Finally, stimulation with all three ligands led to slowing similar to that seen with anti-CD3 together with VCAM-1, demonstrating that the slowing effect observed upon engagement of β1 integrin dominates cell behavior. As a simpler way of expressing these phenotypes, we considered actin flow rates within the lamellipodial region alone (defined as the outer 20% of the IS radius; shaded region in Figure 1B). Within this region, Jurkat T cells spreading on anti-CD3 alone or anti-CD3 + ICAM-1 exhibited actin flow rates of 85–95 nm/s, whereas co-engagement of VCAM-1 (alone or together with ICAM-1) slowed flow to about 15 nm/s (Figure 1E).

Since there are multiple abnormalities in the transformed Jurkat T cell line, we asked if the differential effects of VLA-4 and LFA-1 engagement are recapitulated in primary human T cells. CD4^+^ T cells were purified and expanded from human peripheral blood and transduced with GFP–Lifeact. When stimulated with anti-CD3, these primary human CD4^+^ T lymphoblasts exhibited actin flow rates that were slightly faster than those in Jurkat T cells (Figure 1F; Videos S4–S7 in Supplementary Material). As in Jurkat cells, addition of VCAM-1 to the stimulatory surfaces retarded actin flow. Interestingly, however, primary T cells differed from Jurkat T cells in that costimulation with ICAM-1 retarded actin flow to a similar extent as VCAM-1. A modest additional decrease was observed in cells responding to anti-CD3 together with both integrin ligands.

The differences we observe between Jurkat T cells and primary human CD4^+^ T lymphoblasts can be explained, at least in part, by differences in cell surface integrin expression. Flow cytometric analysis revealed that in comparison with primary T cells, the Jurkat T cells express relatively low levels of LFA-1 (Figure 1C). Indeed, the mean fluorescence intensity of Jurkat T cells was approximately fivefold lower than that of primary T cells. Note that since Jurkat T cells have ~3-fold more surface area than primary human T cells, LFA-1 surface density is approximately 15-fold lower than that of primary T cells. On the other hand, Jurkat T cells constitutively express very high levels of VLA-4, even when compared with primary human CD4^+^ T cells after 7 days of stimulation, a time when their VLA-4 expression is maximal (Figure 1D and data not shown). Moreover, the primary T cell population is more heterogeneous; some cells express high levels of VLA-4 comparable to Jurkat T cells, while others are nearly negative. This probably accounts for the bimodal distribution of actin flow rates observed when these cells engage anti-CD3 and VCAM-1 (Figure 1F). In support of this view, one population of cells (presumably the high VLA-4 expressors) exhibits actin retardation similar to that seen in Jurkat T cells. In any case, these results show that in primary human T lymphoblasts, engagement of either β1 or β2 integrins dramatically slows centripetal actin flow.

### Ligand Mobility Influences Actin Flow

**Figure 2:**
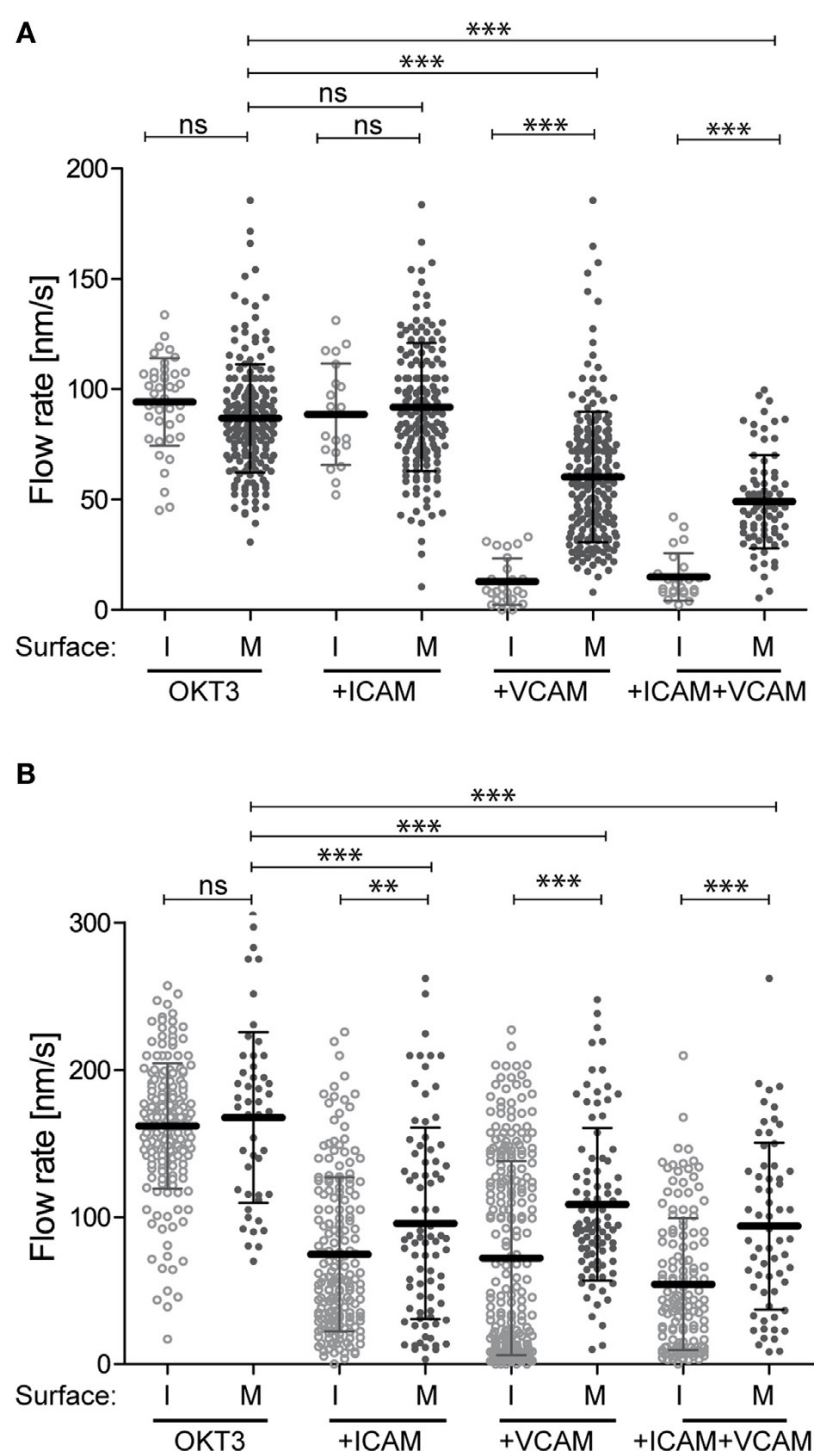
Integrin–ligand mobility influences actin flow. Jurkat T cells **(A)** and primary human CD4^+^ T cell blasts **(B)** were allowed to interact with mobile bilayers functionalized with anti-CD3 (OKT3) alone, together with ICAM-1, VCAM-1, or both. Actin flow rates in the LP region were analyzed as described for Figure 1. Solid dots show flow rates from cells responding to mobile ligand (M). Open dots show data for cells responding to immobile ligands (I). Data for immobile surfaces are from Figure 1, plotted here to facilitate comparison. Error bars show mean ± SD. **p < 0.01, ***p < 0.001.

Engaged integrins could retard the flow of the F-actin network through mechanical coupling (i.e., by exerting drag), by signaling to actin-regulatory proteins, or both. To assess the contribution of mechanical coupling, we asked if the mobility of integrin ligands influences actin flow rates. Studies parallel to those shown in Figure 1 were performed, except that instead of immobilizing anti-CD3, VCAM-1, and ICAM-1 on glass coverslips, these ligands were used to functionalize supported lipid bilayers. Ligands were attached to the lipid head groups so that they were as mobile as the lipids themselves, and FRAP analysis was performed to verify free ligand mobility (data not shown). Figure 2A shows actin flow rates in Jurkat T cells stimulated using mobile ligands, plotted beside rates in cells responding to immobile ligands (from Figure 1E) to facilitate comparison. Flow rates in Jurkat cells responding to immobile and mobile anti-CD3 alone were comparable. As observed with immobile ligands, addition of VCAM-1, but not ICAM-1, slowed actin flow in Jurkat cells. However, on mobile surfaces, VCAM-1 only slowed actin flow to 60 nm/s, as opposed to 15 nm/s on immobile glass surfaces. Primary T cells behaved similarly to changes in ligand mobility (Figure 2B, plotted with data from Figure 1F). Here, costimulation with either ICAM-1 or VCAM-1 retarded actin flow, and the magnitude of retardation was diminished if integrin ligands were presented on mobile surfaces. The observation that slowing of actin flow is maximal when T cells interact with low mobility integrin ligands supports a mechanical model in which ligand-bound integrins engage actin filaments and exert drag forces that oppose centripetal flow. By contrast, the finding that actin flow is slowed significantly even when ligands are freely mobile points to the involvement of more complex biology, since viscous drag should be very low in this experimental condition. Additional analysis will be required to determine the basis of this effect, but it seems likely that biochemical mechanisms involving regulation of actin polymerization are also involved.

### The Effects of Integrin Engagement Are Distributed across the Actin Network

**Figure 3:**
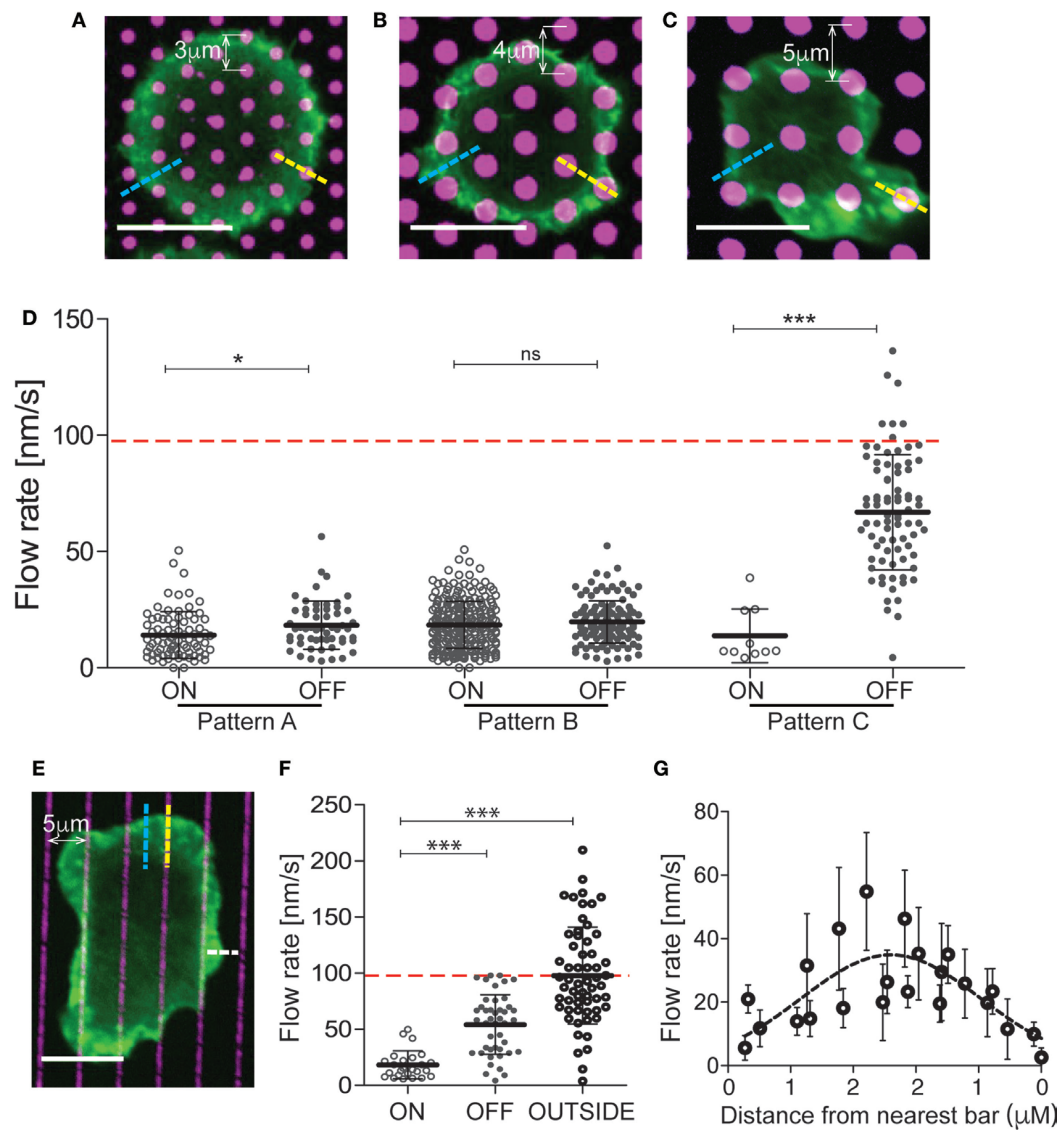
The effects of integrin engagement are distributed across the actin network. Jurkat T cells were allowed to interact with mixed-mobility surfaces bearing patterns of immobilized VCAM-1, surrounded by planar bilayers coated with anti-CD3 and imaged for 4 min. Pattern **(A)** has 1 µm diameter spots with center-to-center distance of 3 µm. Patterns **(B,C)** have 2 µm diameter spots with center-to-center distances of 4 and 5 µm, respectively. **(D)** Actin flow rates were assessed within the lamellipodial region, choosing areas that overlie the immobile spots (on pattern, yellow dashed lines) or bypass them completely (off pattern, blue dashed lines). The red dashed line marks the actin flow on bilayers coated with anti-CD3. **(E)** Jurkat T cells were allowed to interact with surfaces patterned with bars of immobilized VCAM-1 (1 µm wide, 5 µm center-to-center), surrounded by planar bilayers coated with anti-CD3 and imaged for 4 min. **(F)** Actin flow rates were assessed within the lamellipodial region, choosing areas overlying the immobile bars (on pattern, yellow dashed lines), bypass them completely (off pattern, blue dashed lines) or where the free edge of the lamellipodium lies between bars (outside pattern, white dashed line). The red dashed line marks actin flow for cells spread on bilayers coated with anti-CD3. **(G)** Measurements of actin velocity were made parallel to the bars in the space between two bars. Mean ± SD is calculated for measurements made in 20–40 cells for each condition, *p < 0.05, ***p < 0.001. Scale bars = 10 µm.

On the surface of bona fide APCs, MHC Class II molecules are uniformly distributed and freely mobile, while integrin ligands cluster in punctate regions where their mobility is constrained by interaction with the cortical cytoskeleton (38). To model this situation, we generated mixed-mobility surfaces bearing patterns of immobilized integrin ligands surrounded by planar bilayers coated with mobile anti-CD3. Three different patterns varying in spot size, spacing, and geometry were created (Figures 3A–C), and the effect of each pattern on T cell actin flow was tested. To facilitate analysis, these studies were performed using Jurkat T cells, which are larger and exhibit more uniform actin dynamics than primary cells, and using VCAM-1 as the immobilized integrin ligand. Actin flow rates were assessed within the lamellipodial region, choosing areas that maximally overlay immobile VCAM-1 spots (on pattern, yellow dashed lines in Figures 3A–C) or bypass them completely (off pattern, blue dashed lines in Figures 3A–C). Figure 3D shows actin flow rates for each region, in comparison with the response to a uniform mobile bilayer coated with anti-CD3 alone (indicated by a red dashed line). Actin flow was dramatically slowed in T cells interacting with patterns A and B (hexagonal arrays with relatively close spacing). Interestingly, slowing was observed whether measurements were made on or off the pattern, indicating that slowing effects are distributed over the actin network, away from sites of integrin engagement. Actin flow was also strongly slowed in T cells responding to pattern C (a square array with more open spacing), but only for on-pattern measurements. More modest retardation was observed off pattern using this array, where the conduit between integrin spots is significantly wider than in patterns A and B.

To further probe the behavior of the actin network, we analyzed T cells responding to mixed-mobility surfaces where VCAM-1 is organized in bars (Figure 3E). As shown in Figures 3E,F, actin flowed freely in regions where the free edge of the lamellipodium fell outside the pattern (outside, see white line in Figure 3E), was slowed dramatically along the bars (on pattern), and showed intermediate flow rates between bars (off pattern). Measurements made at various points parallel to the bars revealed that flow rates increase with distance from the nearest site of integrin engagement (Figure 3G). Taken together, these results show that the effects of localized integrin engagement are distributed across the actin network, impacting actin dynamics many microns away from the site of integrin engagement. This finding further emphasizes the mechanical nature of integrin effects on the overall actin network.

### Integrin Engagement Modulates TCR Signaling

**Figure 4:**
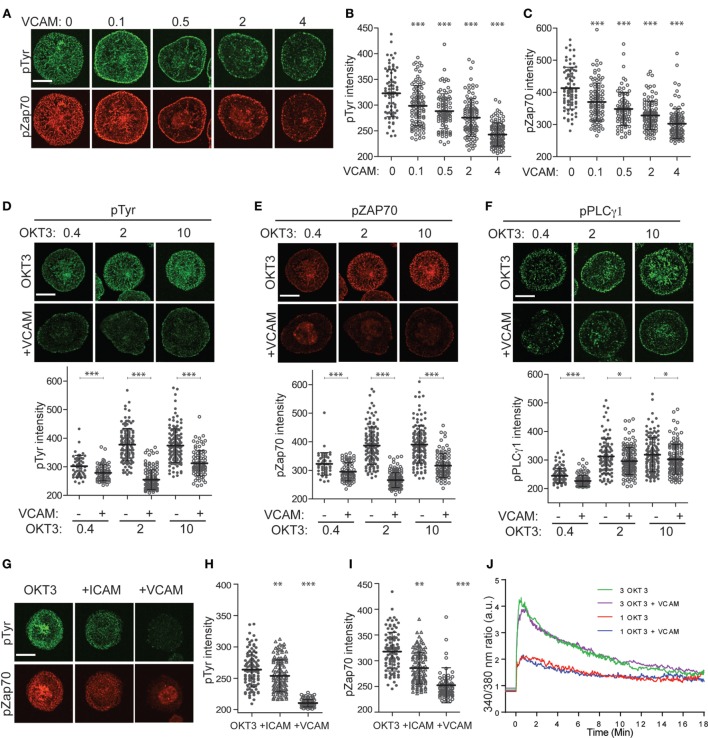
Integrin engagement modulates signaling downstream of the T cell receptor. **(A)** Jurkat T cells were stimulated on coverslips coated with 10 µg/ml OKT3 alone or together with VCAM-1 at varying concentrations (0–4 µg/ml). After 5 min, cells were fixed and stained for phospho-tyrosine (green) and phospho-ZAP70 (Y319, red). **(B)** Quantitative analysis of phospho-tyrosine labeling intensity. **(C)** Quantitative analysis of phospho-ZAP70 labeling intensity. **(D–F)** Jurkat T cells were stimulated on coverslips coated with 0.4, 2, or 10 µg/ml OKT3, alone or together with 2 µg/ml VCAM-1. After 5 min, cells were fixed and stained for phospho-tyrosine **(D)**, phospho-ZAP70 Y319 **(E)** or phospho-PLCγ1 Y783 **(F)**. Quantitative analysis of labeling intensity is shown below each image panel. **(G)** Primary human T cells were stimulated for 5 min on coverslips coated with 10 µg/ml OKT3 alone or together with ICAM-1 or VCAM-1 (each at 4 µg/ml). Cells were fixed and labeled for phospho-tyrosine (green) and phospho-ZAP70 Y319 (red). **(H,I)** Quantitative analysis of labeling intensity from **(G)**. Mean ± SD for 50–60 cells per condition is shown. **(J)** Jurkat T cells loaded with Fura-2 were stimulated on coverslips coated with 1 or 3 µg/ml OKT3, alone or together with 2 µg/ml VCAM-1, and Ca^2+^ responses were monitored by ratiometric imaging. Lines represent averages of 14–23 cells per condition, after alignment of the traces based on the earliest detectable signal over baseline. Lines are artificially extended (before time 0) to better show the starting baseline intensities. Data from one representative experiment (of three) is shown. *p < 0.05; **p < 0.01.***p < 0.001. Scale bars = 10 µm.

To explore the relationship between integrin engagement, actin flow, and TCR signaling, we labeled spreading T cells with antibodies that detect early tyrosine phosphorylation events in the TCR signaling cascade. As shown in Figures 4A–C, Jurkat T cells spreading on anti-CD3 together with increasing doses of VCAM-1 exhibit dose-dependent diminution of overall tyrosine phosphorylation, as well as diminished phosphorylation of ZAP-70 at the activating residue Y319. In previous work, Nguyen et al. showed that VCAM-1 engagement augmented TCR signaling as measured by Ca^2+^ flux and NF-AT activation (39). Since Nguyen et al. used a lower concentration of anti-CD3 than that used here, we considered the possibility that integrin-dependent enhancement of signaling can only be observed under conditions of sub-optimal TCR triggering. To test this, we titered down the dose of anti-CD3 until minimal signaling was observed. In our experimental system, this occurred at 0.4 µg/ml OKT3 (Figures 4D–F). Nonetheless, at this dose of anti-CD3 (and at all other doses tested), engagement of VLA-4 resulted in reduced overall tyrosine phosphorylation, as well as reduced phosphorylation of both ZAP-70 and PLCγ1 at activating sites.

**Figure 5:**
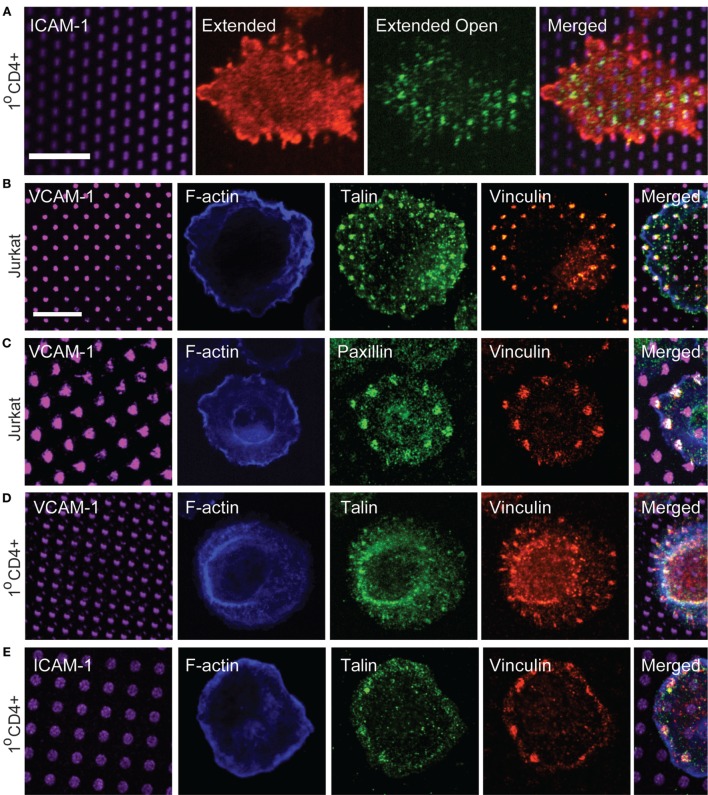
Focal adhesion proteins are recruited to sites of integrin engagement. **(A)** Primary human T cells were stimulated on coverslips patterned with ICAM-1 surrounded with OKT3 for 15 min, and labeled with m24 to detect the active, extended-open conformation of LFA-1, and Kim127 to detect the extended form of LFA-1. **(B,C)** Jurkat T cells were stimulated on VCAM-1 patterns surrounded with anti-CD3 for 15 min. Cells were then fixed and labeled with phalloidin to detect F-actin, and with antibodies to talin, vinculin, and paxillin. **(D,E)** Primary human T cells were allowed to interact with VCAM-1 **(D)** or ICAM-1 **(E)** patterns surrounded with OKT3 for 17 min. Cells were then fixed and labeled with phalloidin to detect F-actin, and with antibodies to talin and vinculin. Far right panels in **(B–E)** show cropped regions in which the four channels have been merged. **(E)** Scale bars = 10 µm.

As shown in Figures 5G-I, integrin engagement also diminished tyrosine phosphorylation in primary human CD4^+^ T lymphoblasts. While the most profound effects were observed after costimulation with VCAM-1, significant diminution in signaling was also observed after costimulation with ICAM-1. Thus, in these cells, engagement of either VLA-4 or LFA-1 diminishes signaling. This is especially important because adhesion at the IS is dominated by LFA-1–ICAM-1 interactions, making this the physiologically relevant receptor–ligand pair.

Our finding that integrin engagement diminishes tyrosine phosphorylation at the IS was surprising, given that integrins are well known to costimulate T cell activation. All of the cells we analyzed were well spread, indicating that they had, in fact, responded to a TCR stimulus. To confirm this, we tested the effect of integrin engagement on Ca^2+^ signaling, the earliest detectable event after TCR triggering. As shown in Figure 4J and Figure S1 in Supplementary Material, stimulation of Jurkat T cells in the presence or absence of VCAM-1 gave identical Ca^2+^ responses, and this result was obtained for two different doses of OKT3. The finding that early Ca^2+^ responses are unaffected by integrin ligands is consistent with the known requirement for initial TCR signaling as a prerequisite for actin polymerization and integrin activation. Taken together, our results are consistent with a model in which the earliest TCR signaling events set actin flow into motion at the IS, thereby fostering additional force-dependent tyrosine phosphorylation, while also activating an integrin-dependent mechanism that governs this mechanotransduction.

### Focal Adhesion Proteins Are Recruited to Sites of Integrin Engagement

Based on our signaling studies, proteins involved in coupling integrins to actin flow should be important modulators of T cell activation. Linkage of integrins to the actin cytoskeleton is best studied in mesenchymal cells, which form well-defined focal adhesions and related adhesive structures. Such structures are not typically observed in T cells, which lack actin stress fibers and generate highly dynamic adhesions associated with low traction forces (33, 34, 41). Canonical focal adhesion proteins, such as vinculin, talin, and paxillin are enriched at the IS (42–44), and in protrusive invadopodium- or podosome-like structures (26, 45–47), but how adhesion complexes are organized at the IS has not been carefully examined. We, therefore, used surfaces bearing patterned integrin ligands and a uniform field of immobilized anti-CD3 to impose structure and ask if focal adhesion proteins are recruited to T cell adhesive contacts. To verify that ICAM-1 patterns correspond to sites of integrin engagement, we first labeled primary human CD4^+^ T lymphoblasts with Kim 127 and m24, antibodies that specifically detect the extended (intermediate affinity) and extended-open (high affinity) conformations of LFA-1, respectively (48, 49). We have previously used these antibodies to analyze T cells spreading on a uniform field of ICAM-1, and shown that the extended conformation of LFA-1 (detected with Kim 127) is enriched in lamellipodial protrusions, while the extended-open conformation (detected with m24) is enriched in an inner ring corresponding to the lamellar region, where actin-dependent forces induce conformational change (23). The distribution of LFA-1 activation intermediates was similar for T cells responding to patterned ICAM-1 (Figure 5A). Consistent with its higher ligand-binding affinity, the extended-open conformation colocalized strongly with spots of ICAM-1.

We next labeled cells with antibodies to talin, vinculin, and paxillin, to ask if these proteins are recruited to T cell adhesive contacts. As shown in Figures 5B,C, Jurkat T cells showed co-localization of talin, vinculin, and paxillin with VCAM-1 patterns. Similar results were obtained with primary human T cells interacting with either VCAM-1 or ICAM-1 patterns (Figures 5D,E), although talin labeling was not as robust in these cells. Labeling with phalloidin confirmed that even when large adhesive contacts are imposed by substrate patterning, these contacts are not associated with actin stress fibers.

**Figure 6:**
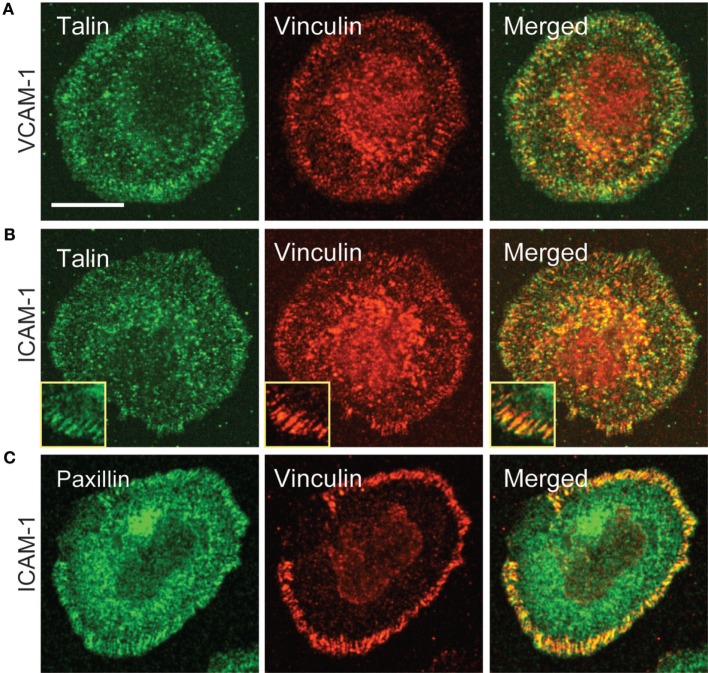
Focal adhesion-like complexes can be detected in T cells spreading on uniform fields of integrin ligand. Primary human T cells were stimulated on coverslips coated with OKT3 and VCAM-1 **(A)** or ICAM-1 **(B,C)** for 17 min, then fixed and stained for talin and paxillin. The inset in **(B)** shows focal adhesion-like structures in a different cell that was stimulated on OKT3 + ICAM-1 for 25 min. Scale bar = 10 µm; inset in B, 8 µm^2^.

Having observed recruitment of canonical focal adhesion proteins to patterns of engaged integrins, we more closely examined cells interacting with a uniform field of integrin ligand to ask if focal adhesion-like complexes could be detected. As shown in Figure 6, primary human T cells spreading on anti-CD3 together with either VCAM-1 or ICAM-1 do sometimes exhibit well-organized adhesive structures containing vinculin, talin, and paxillin. Such structures begin appearing once spreading is maximal (approximately 15 min after addition of cells) and become more prominent over the next 10–20 min (data not shown). In many cases, these structures are elongated and oriented perpendicular to the lamellipodium (inset in Figure 6B). This distribution is consistent with traction mapping studies showing that at late time points of spreading, T cells exert radially arrayed forces on the interacting substrate (33, 35).

### Vinculin and Talin Mediate Integrin-Induced Slowing of the Actin Network

**Figure 7:**
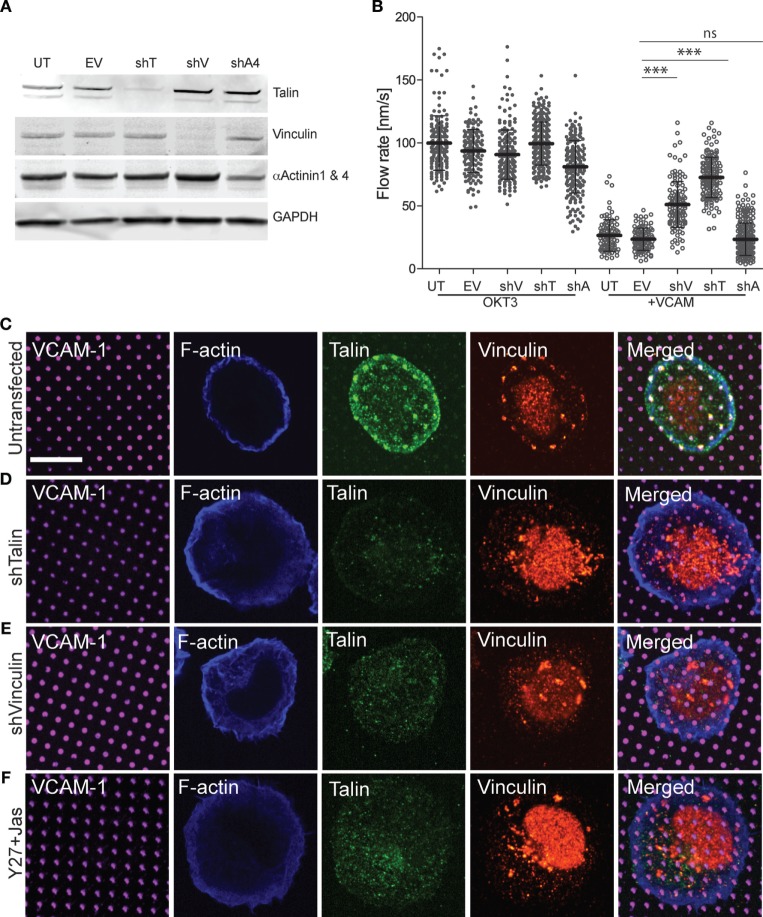
Vinculin and talin couple integrins to actin. **(A)** Jurkat T cells were stably transfected with shRNA vectors targeting talin (shT), vinculin (shV), and α-actinin 4 (shA4). Untransfected cells (UT) and cells expressing the empty shRNA vector (EV) were used as controls. Expression of each protein was tested by Western blotting with the indicated antibodies; the antibody against α-actinin recognizes both the 1 and 4 isoforms. Panels are regions from one blot, adjusted individually to enhance brightness and contrast, using only linear tools. The complete blot is available in Figure S2 in Supplementary Material. **(B)** Cells generated as in **(A)** were stimulated on coverslips coated with OKT3 and VCAM-1, and actin flow rates in the LP region were quantified. Mean ± SD were calculated from measurements made in 10–40 cells for each condition, ***p < 0.001 compared with EV control. **(C–E)** Untransfected **(C)**, talin-suppressed **(D)**, and vinculin-suppressed **(E)** Jurkat T cells were stimulated on coverslips patterned with VCAM-1 surrounded with OKT3 for 15 min, fixed, and labeled as indicated. **(F)** Untransfected Jurkat T cells were treated with Y-27632 and Jasplakinolide to arrest actin flow, and stimulated on coverslips patterned with VCAM-1 surrounded with OKT3. Scale bar = 10 µm.

Talin and vinculin serve as “clutch molecules” that couple engaged integrins to the dynamic actin network (19). Thus, we hypothesized that loss of these molecules would relieve integrin-dependent retardation of actin flow and de-repress TCR signaling. To test this idea, we generated Jurkat T cell lines expressing GFP–actin together with shRNAs targeting talin or vinculin. As controls, we generated a cell line expressing the empty suppression vector, and another expressing control shRNA targeting α-actinin (a focal adhesion protein that is expected to lack clutch activity). Western blot analysis verified that talin and vinculin levels were reduced to less than 10% of control levels (Figure 7A). Only about 50% reduction of α-actinin levels could be achieved, probably reflecting the fact that the shRNA used targets only α-actinin 4, while the available antibody detects both α-actinin 1 and 4. Each of the suppressed cell lines was analyzed by immunofluorescence microscopy using VCAM-1 patterns (Figures 7C–F). Note that only planes near the coverslip are shown, and the vinculin antibody shows significant background staining. As shown in Figures 7D,E, suppression of either talin or vinculin led to loss of both talin and vinculin from VCAM-1 patterns. This result is consistent with evidence from non-hematopoietic cells that these two proteins interact with activated integrins and actin *via* a positive feedback loop (50, 51). Recruitment of vinculin to talin has been shown to involve mechanical force, which unmasks vinculin binding sites within the talin stalk region (52, 53). To ask if this holds true in T cells, cells were treated with the Rho-kinase inhibitor Y-27632 together with the actin stabilizing agent Jasplakinolide, a cocktail that arrests actin flow and relieves force on engaged integrins (23, 29, 30). As shown in Figure 7F, this results in loss of both talin and vinculin from VCAM-1 patterns. Thus, we conclude that talin and vinculin are recruited cooperatively to sites of integrin engagement, *via* a process that depends on ongoing actin flow.

Since suppression of either talin or vinculin resulted in loss of both clutch proteins from sites of integrin engagement, we asked whether this relieves integrin-dependent retardation of actin flow. As expected, all cell lines exhibited comparable actin flow rates when stimulated with OKT3 alone, and retardation of actin flow was observed in untransduced cells and empty vector controls stimulated with OKT3 together with VCAM-1 (Figure 7B; Videos S8–S11 in Supplementary Material). Importantly, suppression of either vinculin or talin significantly reversed the ability of VCAM-1 engagement to retard actin flow. This reversal of integrin-dependent retardation was more pronounced for talin-suppressed cells, which showed actin flow rates approaching those of control cells stimulated with OKT3 alone. This is consistent with models in which talin is the primary linker of integrins to actin, with vinculin reinforcing this connection. Reversal of integrin-dependent retardation was specific to cells deficient for talin or vinculin; cells expressing the α-actinin shRNA behaved like the untransduced and empty vector controls. Taken together, these results show that talin and vinculin work together to couple engaged integrins to the dynamic T cell actin network.

### Coupling of Integrins to the Actin Cytoskeleton Modulates TCR Signaling

**Figure 8:**
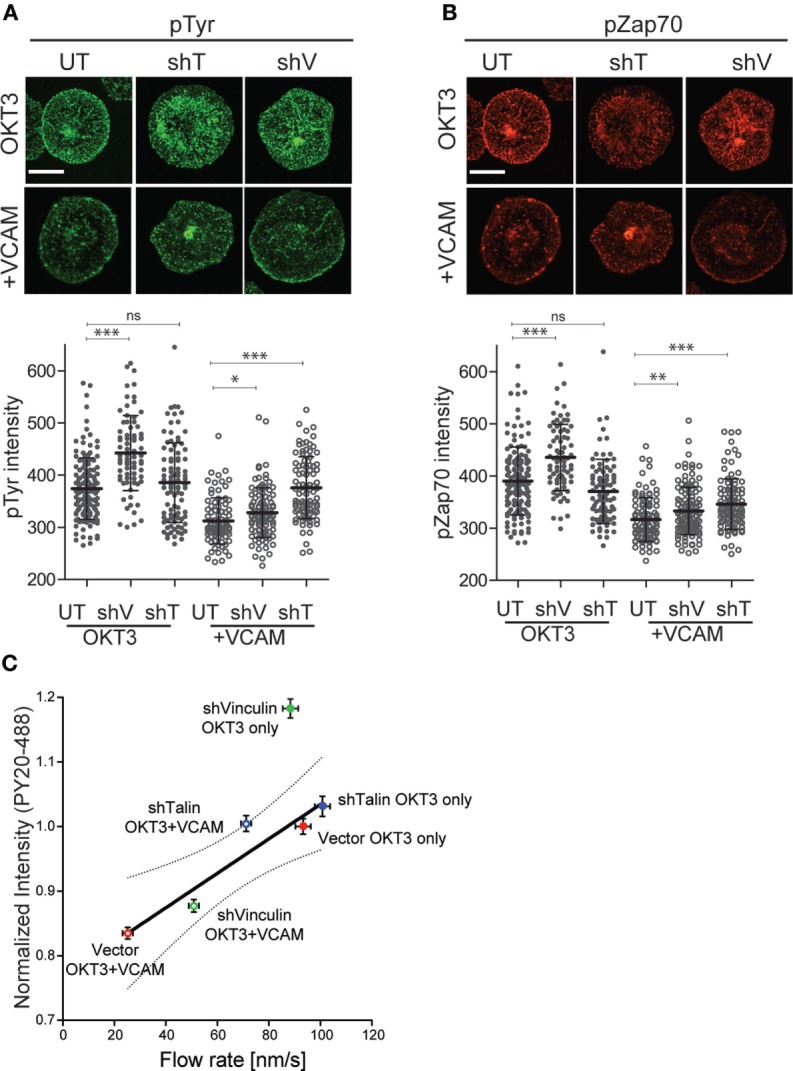
Clutch molecules modulate T cell receptor (TCR) signaling. Jurkat T cells, untransduced (UT), or suppressed for talin (shT) or vinculin (shV) were stimulated on coverslips coated with OKT3 (10 µg/ml) in the presence and absence of VCAM-1 (2 µg/ml). After 5 min, cells were fixed and stained for phospho-tyrosine **(A)** or phospho-ZAP70 (Y319). **(B)** Quantitative analysis of labeling intensity is shown below each image panel. Mean ± SD of measurements from at least 50 cells is shown. *p < 0.05; ***p < 0.001. Scale bars = 10 µm. **(C)** Normalized phospho-tyrosine labeling intensity from **(A)** was plotted against actin retrograde flow rates from Figure 7B. Linear regression analysis showed vinculin-suppressed cells responding to OKT3 alone to be an outlier. Excluding that data point resulted in a strong correlation *R*^2^ = 0.90. Dotted line shows the 95% confidence interval. Data represent means ± SEM from three independent experiments.

Finally, we asked whether in addition to reversing the effects of integrin engagement on actin flow, suppression of talin or vinculin would also relieve inhibition of TCR-dependent tyrosine phosphorylation. As shown in Figure 8A, suppression of either vinculin or talin partially reversed the loss of total tyrosine phosphorylation induced by VCAM-1 engagement. As with actin flow, the effect was greatest for talin-suppressed cells, which showed phospho-tyrosine intensity approaching that of control cells stimulated with anti-CD3 alone. Similar results were obtained for phospho-ZAP70 (Figure 8B), although the reversal was not as complete. One unexpected finding from this study is that vinculin suppression augmented signaling in cells stimulated with anti-CD3 alone. The basis for this effect is unclear, but a similar result was also obtained for ZAP-70. Since vinculin suppression did not accelerate actin flow rates in T cells stimulated on anti-CD3 alone (Figure 7B), this increase in TCR signaling cannot be attributed to changes in actin flow. Thus, vinculin may impact early TCR signaling events in an actin-independent fashion, as well as *via* modulation of integrin-dependent actin dynamics. Additional analysis will be required to address this possibility.

Analysis of the relationship between tyrosine phosphorylation levels and actin flow rates for multiple experimental manipulations revealed a simple, linear correlation (*R*^2^ = 0.63). Linear regression analysis showed that the point representing vinculin-suppressed cells stimulated with OKT3 alone fell outside the 95% confidence interval (data not shown). When this outlying data point was omitted from the analysis, the remaining points correlated strongly (*R*^2^ = 0.90) (Figure 8C). The simplest explanation for this relationship is that actin flow is a key element in determining the magnitude of early signaling events downstream of TCR engagement, and that integrins tune TCR signaling by modulating actin flow.

## Discussion

We showed previously that the cytoskeletal networks of an interacting T cell and APC engage in a sort of tug-of-war at the IS (23, 38). Within the T cell, actin filaments interact with the β2 chain of LFA-1, sweeping it toward the center of the IS. In parallel with this, the DC actin cytoskeleton constrains the mobility of ICAM-1, thereby resisting the forces exerted by the T cell. This mechanical interaction between the two cells fosters integrin conformational changes needed for optimal adhesion and delivery of outside-in signals that costimulate T cell activation. Here, we demonstrate that this tug-of-war has additional consequences that impact T cell signaling. Binding of engaged integrins to the actin cytoskeleton slows the centripetal flow of the T cell actin network. Slowing of actin flow, in turn, diminishes the magnitude of TCR-proximal tyrosine phosphorylation events, an effect that is reversed by suppression of clutch proteins such as talin or vinculin. Taken together, these findings support a model in which the dynamic actin network serves as a mechanical intermediate that coordinates TCR and integrin function at the IS.

Our results clearly show that the rate of centripetal actin flow is directly correlated with the magnitude of tyrosine phosphorylation responses downstream of TCR engagement. These findings are in good agreement with many studies showing that sustained TCR signaling events needed for full T cell activation are dependent on ongoing actin polymerization (16–18, 30). Moreover, they are consistent with models in which TCR signaling is regulated, at least in part, by mechanical force on the TCR and/or associated molecules (21–25). Several mechanisms have been hypothesized to contribute to the mechanical sensitivity of TCR signaling. For instance, the TCR/peptide–MHC interaction displays catch-bond behavior, meaning that the interaction lifetime can actually increase as a function of force applied the TCR (22). In addition, the TCR is thought to undergo force-dependent conformational changes, revealing interaction motifs that are normally hidden under relaxed conditions (54, 55). Although these mechanisms are not mutually exclusive, in both cases the force is likely provided by the actin cytoskeleton (18). In this study, we show that engaged integrins slow actin flow at the IS, similar to how integrin engagement has been shown to retard actin flow in non-hematopoietic cells (19). It, thus, seems likely that, by slowing actin flow, integrin engagement diminishes the total mechanical force on the TCR. In this sense, actin is acting as a true mechanical intermediate between integrins and the TCR, such that integrin regulation of actin dynamics directly influences the mechanotransduction by the TCR. Of course, additional mechanosensitive molecules in the proximal TCR signaling pathway may be similarly affected.

While we find that tyrosine phosphorylation at the IS is diminished by integrin engagement, our analysis of Ca^2+^ signaling shows no sensitivity to integrin ligands. This is actually the expected result, since inside-out signaling induced by initial TCR triggering is needed to drive integrins into the extended, ligand-binding conformation. Moreover, initial Ca^2+^ signals are required to induce actin polymerization at the IS (56, 57), which further promotes integrin activation (23). Thus, until initial TCR signals take place, the T cell is effectively blind to the integrin ligand. Although tyrosine phosphorylation events are needed to induce Ca^2+^ signaling, actin polymerization, and integrin activation, these earliest phosphorylation events are prohibitively difficult for us to quantify by microscopy due to their low magnitude and to the differences in T cell spreading at very early time points. We would predict that they, like the Ca^2+^ signals, would be unaffected by the presence of integrin ligands. Taken together, our results are consistent with a model in which initial contact with the stimulatory surface reflects TCR signals alone. Within less than a minute, however, these signals result in actin flow and high-affinity integrin engagement, such that as the T cell spreads, a negative feedback loop is established that reduces forces on the TCR and dampens tyrosine phosphorylation. Based on this model, one might reasonably expect that sustained Ca^2+^ signals would decay more rapidly in T cells engaging integrin ligands, especially in light of our previous work showing that Ca^2+^ levels drop when actin flow is arrested pharmacologically (30). Since those studies were done without integrin ligands, one interesting possibility is that outside-in signals from integrins help maintain phospho-PLCγ1 at a level that is sufficient to maintain Ca^2+^ responses. In support of this idea, the reduction in PLCγ1 phosphorylation is modest in comparison with the loss of overall tyrosine phosphorylation.

Our finding that tyrosine phosphorylation is reduced by integrin engagement is surprising given that integrin engagement is known to costimulate T cell activation (3–8, 11–13). Most studies of integrin-dependent costimulation have focused on increased proliferation and elevated production of IL-2 and other effector cytokines. Such studies involve long-term T cell–APC interactions, where the ability of integrins to promote sustained cell–cell adhesion undoubtedly plays a large role. Where specific signaling events have been examined, research has focused on enhanced signaling through PI3K, Ras, and Ca^2+^ (6, 58–61). By comparison, tyrosine phosphorylation has not been carefully explored. In a previous study using stimulatory surfaces similar to those used here, Nguyen et al. reported that Jurkat T cells interacting with anti-TCR together with VCAM-1 exhibited higher levels of intracellular Ca^2+^ and increased NF-AT translocation as compared with Jurkat T cells stimulated with anti-TCR alone (39). This is in seeming contradiction to our results. While further experiments will be required to fully reconcile these studies, differences in experimental methods likely account for the discrepancy. In their Ca^2+^ studies, Nguyen et al. used a plate reader and reported responses of the cell population 15–16 min after adding the cells. By contrast, we conducted ratiometric imaging of single cells, which allows analysis of the entire time course, with synchronization to the point when each cell contacts the stimulatory surface. We believe that this strategy provides a more complete and accurate measure of the cell response. Moreover, in the Nguyen study, the largest integrin-dependent increases in T cell activation were observed at very low doses (300 ng/ml) of anti-TCR, where T cell adhesion and spreading was minimal in the absence of integrin ligand. Interestingly, when Nguyen et al. analyzed signaling at 10 µg/ml OKT3, where cells were well spread even in the absence of integrin ligand, integrin engagement induced only a very small increase in NF-AT activity and actually diminished Ca^2+^ responses. For tyrosine phosphorylation studies, we tested low doses of anti-TCR similar to those used by Nguyen et al. in our dose–response experiments, but we chose to analyze phosphorylation only in well-spread T cells. This was done intentionally to minimize differences in TCR engagement, and focus our analysis on T cells responding to surface-bound stimuli where mechanobiology comes into play. Finally, Nguyen et al. labeled cells for phospho-tyrosine and noted that integrin engagement led to a shift in phospho-tyrosine-positive microclusters toward the cell periphery (an effect that we see as well). However, quantitative analysis of tyrosine phosphorylation was not performed in that study. Thus, taking into account our results and those of Nguyen et al., we propose that integrin ligation blunts tyrosine phosphorylation but sustains contact duration and promotes other late signaling events needed for full T cell activation. Indeed, mathematical modeling of weak vs. strong agonists shows that feedback mechanisms balance TCR signal strength, centripetal microcluster flow, and duration of signaling. Our data suggest that integrins may participate in this regulatory loop. Future studies will be directed at testing this mechanism for integrin-dependent costimulatory signaling.

Most of what is known about focal adhesions and related adhesive structures comes from studies in mesenchymal cells, which exert relatively strong traction forces and form actin stress fibers when grown on stiff substrates (62–64). Since T cells exert very weak traction forces and lack stress fibers, the extent to which T cell adhesive contacts resemble focal contacts has been unclear. At a gross level, recruitment of focal adhesion proteins to the IS is well documented. Indeed, talin was one of the proteins first used to define the ring-like pSMAC region of the IS (42, 65). Vinculin is also recruited to the IS, and FRET studies and biochemical analysis show that a complex containing WAVE2, Arp2/3 vinculin, and talin forms upon TCR engagement (43). We now show that engagement of either β1 or β2 integrins in T cells induces the recruitment of canonical focal adhesion proteins, including talin, vinculin, and paxillin. Moreover, we find that these proteins are assembled in familiar ways, i.e., talin and vinculin serve as “clutch molecules” that connect integrins to actin filaments, whereas α-actinin is associated with the complex but does not serve as a direct linker. As in mesenchymal cells, recruitment of talin and vinculin depends on forces exerted by ongoing actin polymerization (51); if actin flow is arrested pharmacologically, both proteins dissociate from T cell adhesive sites. This finding supports and extends earlier work showing that suppression of WAVE2, which disrupts lamellipodial actin polymerization in T cells, leads to loss of both talin and vinculin from the IS (43). Finally, in an effort to reconcile studies showing that Jurkat T cells suppressed for vinculin fail to recruit talin to the IS (43) and murine T cells lacking talin fail to recruit vinculin to the IS (66), we conducted side-by-side analysis using our patterned surfaces to restrict analysis to sites of integrin engagement. We find that talin and vinculin are interdependent on one another for recruitment to adhesive contacts sites, since cells suppressed for talin fail to recruit vinculin, and *vice versa*.

Although T cells lack prominent focal adhesion-like structures, we show that such structures can be observed in cells that have been spreading on glass surfaces for prolonged times. In T cells interacting with APCs, which have a softer surface and more mobile ligands, it seems unlikely that identifiable focal adhesions are ever formed. Instead, there is evidence that the same proteins are organized into structures with additional, higher order, complexity. Parallels have been noted between the overall bullseye structure of the IS and the architecture of podosomes (46). Podosome-like structures with actin-rich cores surrounded by a ring of integrins and talin have been observed at T cell–endothelial contacts (45) and similar structures may be present at the IS (26, 36). In addition, Hashimoto-Tane and Saito recently demonstrated the formation of micron-sized structures they term “micro-synapses,” which have a central region enriched in the TCR and an outer ring comprised of LFA-1 and associated focal adhesion proteins such as paxillin and Pyk2 (47). Understanding the relationship between these and similar structures such as microvilli (36), and parsing out how these all contribute to T cell activation, is an area of intensive investigation.

Our studies show that integrin linkage to the actin cytoskeleton modulates actin flow rates, and that actin flow rates are tightly linked to TCR signal strength. Thus, it stands to reason that clutch molecules like talin and vinculin will prove to be important control points for T cell responses, particularly where mechanotransduction is involved. Indeed, Wernimont et al showed that talin-deficient T cells proliferate efficiently in a contact-independent setting, but show severe defects in proliferation to antigen-bearing APCs (66). These proteins also seem to play a key role in regulating the balance between lymphocyte migration and IS formation, a process that has been described as synapse–kinapse transition (67). When migrating along the APC surface, T cells lacking talin fail to stop properly, despite clear evidence of TCR engagement and signaling (66). Similarly, dysregulation of antigen-dependent stopping has been observed in B cells lacking vinculin (68). The resulting changes in lymphocyte–APC interaction are likely to have far-reaching effects. For example, loss of talin has recently been found to perturb regulatory T cell homeostasis (69). With respect to costimulatory signaling, molecules such as Rap1, FAK, and Pyk2 that regulate the function of clutch proteins may be involved. In keeping with this idea, Pyk2 is important for integrin-dependent costimulation of CD8+ T cells (70). Such molecules could also play a role in crosstalk between costimulatory pathways. For example, Rap1, which plays a key role in integrin activation, is down-modulated by signaling from CD28 (71, 72).

Several important questions remain about how integrins interact with the TCR *via* the actin cytoskeleton. First, although we show for the first time that both β1 and β2 integrins slow actin flow, β1 integrin engagement often has a more profound effect. It is not clear whether this is due to differences in integrin expression levels or ligand-binding affinity, or if there are molecular differences in the clutch protein machinery. Another open question concerns the mechanisms through which engaged integrins retard actin flow. Our data show that retardation is greatest when integrin ligands are immobilized, pointing to a mechanism involving physical drag. Such a mechanism is almost certainly important *in vivo*, where integrin ligands are attached to the extracellular matrix or where their mobility within endothelial or APC membranes is constrained by attachment to the cortical cytoskeleton (38). Interestingly, however, we observe substantial slowing of actin flow even when integrin ligands are presented on mobile bilayers, where drag should be minimal because ligand mobility is high and viscosity is very low. Though membrane proteins rarely show this high level of mobility in *bona fide* cellular membranes, this experimental system reveals the contribution of another process, which cannot be attributed solely to mechanical drag. In keeping with this, suppression of talin or vinculin only partially reverses integrin-dependent actin retardation; even in talin-suppressed cells, which show the largest effect, actin flow rates never reach the rates observed in the absence of integrin ligand. Together, these observations point to a second, clutch-independent mechanism, involving signaling events that control the rate of actin polymerization. The relevant pathway remains to be defined, but it most likely involves modulation of one or more Rho GTPases (73–75).

Increasingly, it is becoming clear that mechanical force is essential for receptor signaling at the IS, and that the actin cytoskeleton serves an integrative role in this context. Going forward, it will be important to tease apart the relevant biochemical and biomechanical pathways though which individual receptors control, and are controlled by, actin dynamics, in order to develop a full understanding of receptor crosstalk at the IS.

## Materials and Methods

### General Reagents, Antibodies, and Recombinant Proteins

Unless otherwise noted, reagents were purchased from Sigma-Aldrich. Y-27632 and Blebbistatin were from EMD Millipore. Jasplakinolide, Alexa Fluor 568-phalloidin, and Alexa Fluor 488-Phalloidin were from Thermo Fisher, and CF405-Phalloidin was from Biotium. Unless otherwise specified, antibody labeling kits (Alexa Fluor 488, 568, 594, and 647) were from Thermo Fisher. Streptavidin was from Jackson ImmunoResearch Laboratories.

The following antibodies were used for immunofluorescence microscopy: rabbit anti-vinculin (Cat. #73412) was from Abcam, and rabbit anti-paxillin (Cat. # sc-5574) was from Santa Cruz Biotechnology. Talin was detected with mouse monoclonal 8D4 (Sigma), after direct conjugation to CF488 using Mix-n-Stain antibody labeling kit (Biotium). Phospho-tyrosine was detected with PY20 (Santa Cruz; #sc-508), directly conjugated to AF-488. Phosphorylated ZAP70 (Y319) and phospholipase C gamma 1 (PLCγ1, Y783) were detected with rabbit polyclonal antibodies from Santa Cruz (#sc-12946-R) and Cell Signaling (#2821), respectively. Secondary antibodies (donkey anti-Rabbit AF-488 and AF-568) were from Thermo Fisher.

For immunoblotting, total α-actinin (1 and 4) was detected using Sigma anti-α-actinin 1 (Cat. # HPA006035). Talin was detected using 8D4, and vinculin was detected with the mouse monoclonal hVin-1 (Sigma), or the Abcam rabbit polyclonal. For flow cytometry, anti-human CD11a–FITC (BD Pharmingen Cat. #55383) was used to label LFA-1, while anti-human CD29-APC (BD Pharmingen # 559883), was used to label VLA-4. Detection of LFA-1 activation intermediates was performed using monoclonal antibodies Kim127 (hybridoma from ATCC), and m24 (Abcam). Kim127 detects both the extended and extended-open conformations of LFA-1, whereas m24 specifically binds to the high-affinity extended-open conformation (48, 49, 76–79).

Artificial stimulatory surfaces were generated using the following reagents: goat anti-human IgG (Fc specific) was from Fitzgerald. Mouse anti-human CD3 (OKT3) was from BioXCell, biotinylated OKT3 was from eBioscience, and human ICAM-1-Fc and VCAM-1-Fc chimeras were from R&D Systems. His-tagged human VCAM-1 Fc was from Sino Biological Inc., and His-tagged human ICAM-1 Fc was purified from cells as previously described (80). For patterning, ICAM-1 and VCAM-1 were labeled with AF-647 using a microscale labeling kit (Invitrogen).

### Cell Culture

The human Jurkat T lymphoma cell line stably expressing GFP–actin (81) was grown in RPMI 1640 containing Penicillin/Streptomycin, Glutamax (all from Gibco Thermo Fisher) and 5% FBS/5% newborn calf serum (Atlanta Biological) at 37°C in 5% CO_2_. Primary human peripheral blood CD4^+^ T cells were obtained without donor identifiers from the Univ. of Pennsylvania’s Human Immunology Core under an Institutional Review Board approved protocol. Lymphoblasts were generated by activation with human T-Activator CD3/CD28 magnetic beads (Dynabeads, Life Technologies) in RPMI supplemented with 10% FBS, 1% GlutaMAX, Penicillin/Streptomycin and 50 U/ml of human rIL-2 (obtained through the AIDS Research and Reference Reagent Program, Division of AIDS, National Institute of Allergy and Infectious Diseases, National Institutes of Health; human rIL-2 from M. Gately, Hoffmann-LaRoche). T lymphoblasts were cultured at 37°C in 5% CO_2_. Beads were magnetically removed on day 7 after initial stimulation, and cells were then cultured for an additional day in the presence of 10 U/ml of IL-2.

### Plasmids and Transduction

TRC shRNA targeting constructs in pLKO.1-puro were obtained from the Univ. of Pennsylvania High Throughput Screening Core. For each target protein, at least five shRNAs were tested, and the clones yielding the best suppression were selected for generation of stably suppressed cell lines. Optimal targeting constructs were as follows: shTalin: CCCAGAGTATTAACGCTCCAA; shVinculin: CGGTTGGTACTGCTAATAAAT; shα-Actinin 4: GCCACACTATCGGACATCAAA. Recombinant lentivirus was generated in 293T cells co-transfected using the calcium phosphate method with each DNA of interest (in pLX301 for expression or pLKO.1-puro for suppression), together with psPAX2 and pMD2.G. Medium was exchanged after 18 h, and supernatant harvested 24 h later was immediately used to transduce T cells.

Primary human CD4^+^ T cells expressing GFP–Lifeact (82) were generated by lentiviral transduction as detailed previously (23). Briefly, cells were induced by spin infection with lentivirus on day 1 after activation with human T-Activator beads. Lentivirus-containing media was replaced with T cell culture media, and after 3 days, puromycin was added to a concentration of 2 µg/ml. Activator beads were removed at day 7 after activation, and cells were used at days 8–9. GFP–actin Jurkat T cells stably suppressed for talin, vinculin, and α-actinin were generated similarly, but without activation. Suppression was monitored by Western blotting throughout the study.

### Western Blotting

Cells were lysed in 1% NP-40 (IGEPAL), 50 mM Tris, pH 8.0, 150 mM NaCl, 5 mM KCl, and 5 mM MgCl_2_, 10 mM NaF, 1 mM NA_3_VO_4_, and complete protease inhibitor cocktail (Roche) for 20 min at 4°C. Lysates were diluted in NuPage sample buffer and separated on NuPage 4–12% Gradient gels (Thermo Fisher). After transferring to nitrocellulose membranes and blocking with 0.5× blocking buffer (Licor Biosciences), proteins were probed with the indicated primary antibodies followed by appropriate secondary antibodies, and then imaged using an Odyssey fluorescence scanner (Li-Cor Biosciences). Images were captured within the linear range for each probe, and sample intensities were normalized to GAPDH staining.

### Flow Cytometry

T cells were washed and suspended in FACS buffer (PBS, 5% FBS, 0.02% NaN_3_, 1 mM EDTA) containing appropriate antibodies. After washing, cells were analyzed on an LSRII flow cytometer (BD Biosciences). Analysis was carried out using FlowJo (Tree Star).

### Preparation of Stimulatory Surfaces

Stimulatory surfaces were prepared as detailed in Ref. (40). For ligands immobilized on glass surfaces, coverslips (for fixed cell studies) or Sticky-Slide VI 0.4 chambers (Ibidi, for live cell studies) were coated with 10 µg/ml OKT3 for 2 h at 37°C or overnight at 4°C, washed, and incubated with 2 µg/ml ICAM-1 Fc and/or VCAM-1 Fc for 2 h at 37°C. For studies using supported planar lipid bilayers, lipids (DOPC, DSPE-PEG2000-biotin, and DOGS-NTA nickel salt; Avanti Polar Lipids, Inc.) were reconstituted in chloroform at 89.9:0.1:10 mol%, dried and then hydrated in PBS. Lipids were sonicated, and then passed through a 50-nm pore membrane using a mini-extruder (Avanti Polar Lipids, Inc.) to generate small unilamellar vesicles. 25 × 75-mm glass coverslips were cleaned using Piranha solution (3:1 ratio of sulfuric acid and 30% hydrogen peroxide) or Nochromix (Godax Laboratories Inc.), washed with distilled water, air dried, and applied to Sticky-Slide VI 0.4 chambers. Small unilamellar vesicles were added to the chambers to cover the exposed glass surface for 15 min, after which chambers were rinsed with PBS, and incubated with His-tagged ICAM-1 or VCAM-1 (2 µg/ml), followed by 1 µg/ml streptavidin for 15 min. After rinsing, chambers were then incubated with 10 µg/ml OKT3-biotin for 15 min. Chambers were rinsed and left in L-15 medium supplemented with 2 mg/ml d-glucose (imaging medium). Lipid bilayers were used for imaging studies on the same day.

To prepare mixed-mobility stimulatory surfaces, PDMS stamps with patterns were cast on fabricated silicon wafers. Goat anti-human IgG antibodies (50 µg/ml) were deposited onto the stamps, washed with distilled water, and dried. Patterns were then stamped onto Nochromix-cleaned coverslips for 1 min, after which the coverslips were dried and adhered to Sticky-Slide VI 0.4 chambers. Chambers were then incubated with small unilamellar vesicles containing biotinylated lipids (DOPC:DSPE-PEG2000-biotin 99.9:0.1) as described above. After thorough washing with PBS, chambers were incubated with ICAM-1 Fc or VCAM-1 Fc (each labeled with AF-647), streptavidin and OKT3-biotin, maintained in imaging medium, and used the same day.

For fixed cell studies on patterned surfaces, Nochromix-cleaned coverslips were stamped directly with 20 µg/ml VCAM-1 or ICAM-1 labeled with AF-647 as described above. The stamped coverslips were then coated with 10 µg/ml OKT3 for 2 h at 37°C or overnight at 4°C, washed once with PBS, and twice with imaging medium prior to use.

### Fluorescence Microscopy

Fixed and living cells were imaged on a Perkin Elmer Ultraview spinning disk confocal system, using a 63x Plan Apo, 1.4N.A. objective mounted on a Axiovert 200M microscope platform (Carl Zeiss) equipped with an Orca ER CCD camera (Hamamatsu). Images were acquired and analyzed using Volocity v. 6.3 imaging software (Perkin Elmer).

For fixed cell imaging, T cells were harvested, resuspended in imaging medium, and pipetted onto stimulatory coverslips. Cells were allowed to spread for the indicated times at 37°C, after which they were washed in PBS and fixed in 3% paraformaldehyde/PBS. After quenching with 50 mM NH_4_Cl, cells were permeabilized with 0.1% Tx-100/PBS and blocked with PBS/0.01% saponin/0.25% fish skin gelatin. Cells were labeled with primary antibodies followed by secondary antibodies, as indicated. Primary antibodies against pZAP70, pTyr, and pPLCγ1 were incubated overnight at 4°C, otherwise antibody labeling was for 1 h at RT. Because both the stimulatory antibody OKT3 and anti-talin antibody 8D4 are mouse monoclonals, anti-talin was directly conjugated to CF488 to avoid crossreactivity with the secondary antibody. After antibody labeling, F-actin was visualized with fluorescent phalloidin, nuclei were stained with either 0.1 µg/ml DAPI (Sigma), or 5 mM Draq5 (eBioscience), and coverslips were mounted onto slides with Mowiol 4-88 (Sigma). Labeling with conformation-specific integrin antibodies was performed as described previously (23). Kim127 was conjugated to AF-594, and LFA-1 was directly conjugated to AF-488. Because the epitope recognized by m24 is destroyed by fixation, cells were labeled with this antibody for 5 min prior to fixation. Cells were then fixed and processed as described above.

Live-cell imaging was conducted as previously described (30, 40). Ibidi μ-Slide VI chambers coated with stimulatory ligands were filled with imaging medium and equilibrated at 37°C. Cells were resuspended at 1 × 10^6^/ml in imaging medium and pipetted into each well. Imaging began 3–5 min after cell addition to allow the cells to contact and spread on the chamber bottoms. Three image planes were captured through focus in 0.25 µm increments and collected every 1–2 s for 4 min. Emission discrimination filters were used for multicolor imaging. During the next 30 min, a series of 4–5 movies, consisting of 5–8 cells per field, were collected for 4 min each. Fields to be analyzed were selected at random, and analysis was restricted to well-spread cells in which actin behavior was clearly visible. Actin flow was measured at 9–15 sites per cell, in 20–60 total cells accumulated from at least three independent experiments.

### Image Analysis

Kymographic analysis was performed using Volocity v. 6.3, as detailed in Ref. (30, 40). Briefly, kymographs were generated by drawing a ray from the center of IS to its periphery. Actin flow rates were calculated based on the angle of deflection from the vertical direction. IS radius values from individual cells were normalized to 1, and instantaneous velocities were plotted as a function of the IS radius and grouped into 10 equally spaced bins. Flow rates were calculated in the lamellipodium region, defined as the normalized cell radius in the range of 0.8–1. For actin flow analysis on patterned surfaces, kymographs were generated by choosing areas that maximally intersected the immobile spots (on pattern), bypassed them completely (off pattern), or lay outside the patterned region (outside).

### Quantitative Analysis of Tyrosine Phosphorylation

To measure tyrosine phosphorylation, the relative amounts of pTyr, pZAP-70, and pPLCγ1 were determined by labeling with relevant phospho-specific antibodies, and then measuring the fluorescence intensity at the IS. Briefly, T cells were allowed to settle and spread onto stimulatory surfaces for 5 min to activate signaling, and fixed for 5 min before labeling. Sixteen fields were imaged near the plane of the coverslip, each containing multiple cells. In each case, a *z*-series containing 3-planes spaced 0.25 µm apart was captured. The integrated signal intensity from the rendered stack was measured using Volocity 6.3 by manually drawing a ROI around each cell. Only intact cells with a well-spread morphology were analyzed. Data represent mean ± SD from 50 to 60 cells per condition.

### Calcium Imaging

Jurkat T cells were loaded with 2 µM Fura-2 acetoxymethyl ester (Thermo-Fisher) in a buffer solution containing 145 mM NaCl, 4.5 mM KCl, 2 mM CaCl_2_, 1 mM MgCl_2_, 10 mM glucose, 10 mM HEPES, 2 mM glutamine, and 2% fetal bovine serum for 15 min at room temperature. For imaging, cells were washed and resuspended in buffer solution. Stimulatory surfaces were prepared as described above. Ca^2+^ responses were imaged using a Zeiss Plan-Neofluor 40× oil objective (N.A. 1.3) on a Zeiss Axiovert 200 M inverted microscope equipped with an MS-2000 automatic stage (Applied Scientific Instruments) and a 37°C environmental chamber. A Lambda DG-4 Xenon light source (Sutter) was used for rapid transition between 340 and 380 nm excitation. Images were collected for 30 min at 1 to 2-s intervals using Slidebook 6.0 software (Intelligent Imaging Innovation). Analysis of Ca^2+^ data was done using ImageJ software (NIH, Bethesda, MD, USA). Ca^2+^ signal was analyzed by plotting the emission ratio of 340/380-nm excitation for each cell. Individual plots were shifted to correct for different touch down times and an average plot was created for each condition.

### Statistical Analysis

Statistical analysis was performed using Microsoft Excel and Prism software. Unless otherwise indicated, statistical significance was calculated using Student’s *t-*test for unpaired samples, and data are presented as mean ± SD.

## Author Contributions

KJ, EW, NR, and DB conceived and designed the experiments, acquired and interpreted data, and drafted and revised the manuscript. VC acquired and interpreted data and assisted with manuscript preparation. JB and TB conceived and interpreted experiments, and JB drafted and revised the manuscript. All authors approved the final version of the manuscript and all agree to be accountable for the accuracy and integrity of their work.

## Conflict of Interest Statement

The authors declare that the research was conducted in the absence of any commercial or financial relationships that could be construed as a potential conflict of interest.

## Supplementary Material

Video S1 Jurkat T cells expressing GFP–actin were imaged by spinning disk confocal microscopy while spreading on glass coverslips coated with anti-CD3 alone. Rendered stacks of three images planes are played back at 20× real time. Scale bar = 10 µm.

Video S2 Jurkat T cells expressing GFP–actin were imaged by spinning disk confocal microscopy while spreading on glass coverslips coated with anti-CD3 + VCAM-1. Rendered stacks of three images planes are played back at 20× real time. Scale bar = 10 µm.

Video S3 Jurkat T cells expressing GFP–actin were imaged by spinning disk confocal microscopy while spreading on glass coverslips coated with anti-CD3 + ICAM-1. Rendered stacks of three images planes are played back at 20× real time. Scale bar = 10 µm.

Video S4 Primary human CD4 + T cells expressing GFP–Lifeact were imaged by spinning disk confocal microscopy while spreading on glass coverslips coated with anti-CD3 alone. Rendered stacks of three images planes are played back at 20× real time. Scale bar = 10 µm.

Video S5 Primary human CD4+ T cells expressing GFP–Lifeact were imaged by spinning disk confocal microscopy while spreading on glass coverslips coated with anti-CD3 + VCAM-1. Rendered stacks of three images planes are played back at 20× real time. Scale bar = 10 µm.

Video S6 Primary human CD4+ T cells expressing GFP–Lifeact were imaged by spinning disk confocal microscopy while spreading on glass coverslips coated with anti-CD3 + ICAM-1. Rendered stacks of three images planes are played back at 20× real time. Scale bar = 10 µm.

Video S7 Primary human CD4+ T cells expressing GFP–Lifeact were imaged by spinning disk confocal microscopy while spreading on glass coverslips coated with anti-CD3 + ICAM-1 + VCAM-1. Rendered stacks of three images planes are played back at 20× real time. Scale bar = 10 µm.

Video S8 Jurkat T cells expressing GFP–actin and an empty shRNA control vector were imaged by spinning disk confocal microscopy while spreading on glass coverslips coated with anti-CD3 alone. Rendered stacks of three images planes are played back at 20× real time. Scale bar = 10 µm.

Video S9 Jurkat T cells expressing GFP–actin and an empty shRNA control vector were imaged by spinning disk confocal microscopy while spreading on glass coverslips coated with anti-CD3 + VCAM-1. Rendered stacks of three images planes are played back at 20× real time. Scale bar = 10 µm.

Video S10 Jurkat T cells expressing GFP–actin and suppressed for talin were imaged by spinning disk confocal microscopy while spreading on glass coverslips coated with anti-CD3 + VCAM-1. Rendered stacks of three images planes are played back at 20× real time. Scale bar = 10 µm.

Video S11 Jurkat T cells expressing GFP–actin and suppressed for vinculin were imaged by spinning disk confocal microscopy while spreading on glass coverslips coated with anti-CD3 + VCAM-1. Rendered stacks of three images planes are played back at 20× real time. Scale bar = 10 µm.

Figure S1 Single cell Ca_2+_ response data used to generate Figure 4J. Jurkat T cells loaded with Fura-2 were stimulated on coverslips coated with 1 or 3 μg/ml OKT3, alone or together with 2 μg/ml VCAM-1, and Ca_2+_ responses were monitored by ratiometric imaging. Individual cell responses (each represented as a colored trace) were aligned to time zero based on the earliest detectable signal over baseline. Black lines represent the population averages. Traces were artificially extended (before time 0) to better show the starting baseline intensities. Data from one representative experiment (of three) is shown. **(A)** 1 μg/ml OKT3 only, n = 17. **(B)** 1 μg/ml OKT3 and 2 μg/ml VCAM-1, n = 21. **(C)** 3 μg/ml OKT3 only, n = 14. **(D)** 3 μg/ml OKT3 and 2 μg/ml VCAM-1, n = 23.

Figure S2 The entire immunoblot used to generate Figure 7A. Lysates from Jurkat T cells untransduced or stably expressing the indicated lentiviral constructs were separated by SDS-PAGE and probed by immunoblotting with the indicated antibodies, confirming successful knockdown of Talin, Vinculin and alpha-Actinin 4. UT—untransduced, EV—empty vector, shT—shRNA to Talin, shV—shRNA to Vinculin, shA 4—shRNA to alpha-Actinin 4.
