# Supplementary material for: Integrins Modulate T Cell Receptor Signaling by Constraining Actin Flow at the Immunological Synapse
Source: Front Immunol. 2018 Jan 18;9:25. doi: 10.3389/fimmu.2018.00025 (PMC5778112; doi:10.3389/fimmu.2018.00025)
Supplement: Figure S1 — Single cell Ca2+ response data used to generate Figure 4J. Jurkat T cells loaded with Fura-2 were stimulated on coverslips coated with 1 or 3 μg/ml OKT3, alone or together with 2 μg/ml VCAM-1, and Ca2+ responses were monitored by ratiometric imaging. Individual cell responses (each represented as a colored trace) were aligned to time zero based on the earliest detectable signal over baseline. Black lines represent the population averages. Traces were artificially extended (before time 0) to better show the starting baseline intensities. Data from one representative experiment (of three) is shown. (A) 1 μg/ml OKT3 only, n = 17. (B) 1 μg/ml OKT3 and 2 μg/ml VCAM-1, n = 21. (C) 3 μg/ml OKT3 only, n = 14. (D) 3 μg/ml OKT3 and 2 μg/ml VCAM-1, n = 23. [file image_2.PDF]

Supplementary Material

Integrins modulate TCR signaling by constraining actin flow at the immunological synapse

Katarzyna I. Jankowska, Edward K. Williamson, Nathan H. Roy, Daniel Blumenthal, Vidhi Chandra, Tobias Baumgart[2], and Janis K. Burkhardt*

***Correspondence:** Janis K Burkhardt: jburkhar@pennmedicine.upenn.edu

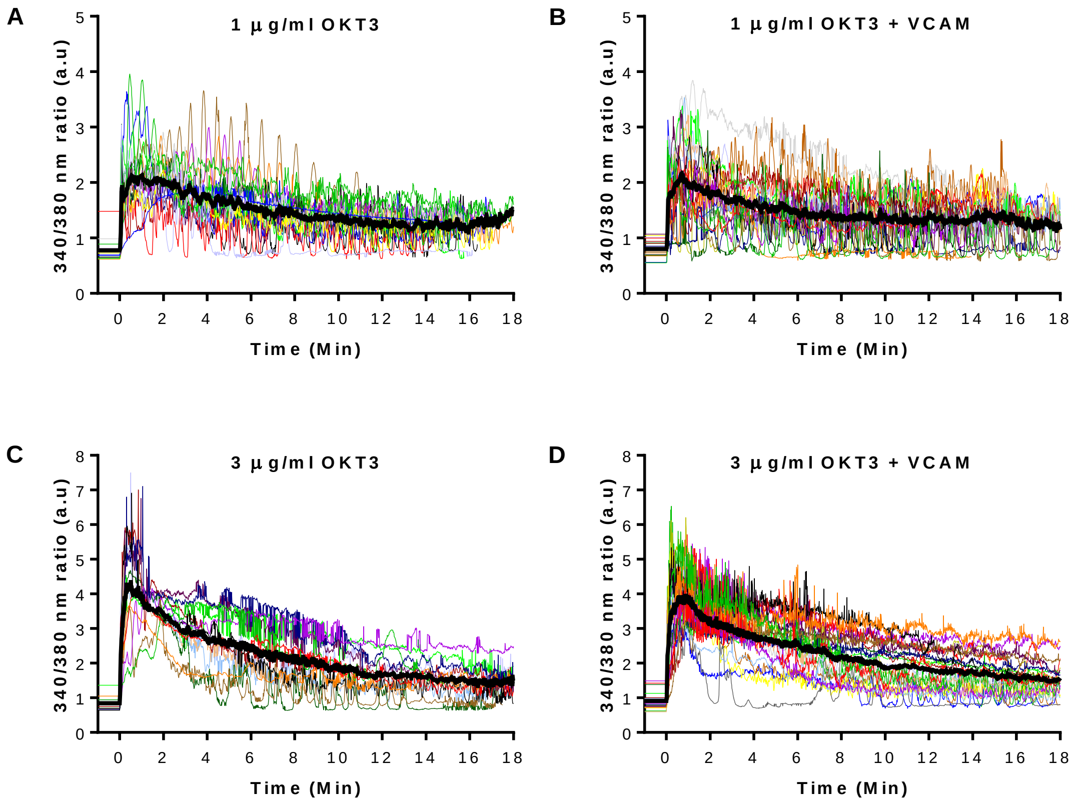

Supplementary Figure 1. Single cell Ca^{2+} response data used to generate Figure 4J. Jurkat T cells loaded with Fura-2 were stimulated on coverslips coated with 1 or 3 µg/ml OKT3, alone or together with 2 µg/ml VCAM-1, and Ca^{2+} responses were monitored by ratiometric imaging. Individual cell responses (each represented as a colored trace) were aligned to time zero based on the earliest detectable signal over baseline. Black lines represent the population averages. Traces were artificially extended (before time 0) to better show the starting baseline intensities. Data from one representative experiment (of three) is shown. (A) 1 µg/ml OKT3 only, n=17. (B) 1 µg/ml OKT3 and 2 µg/ml VCAM-1, n=21. (C) 3 µg/ml OKT3 only, n=14. (D) 3 µg/ml OKT3 and 2 µg/ml VCAM-1, n=23.

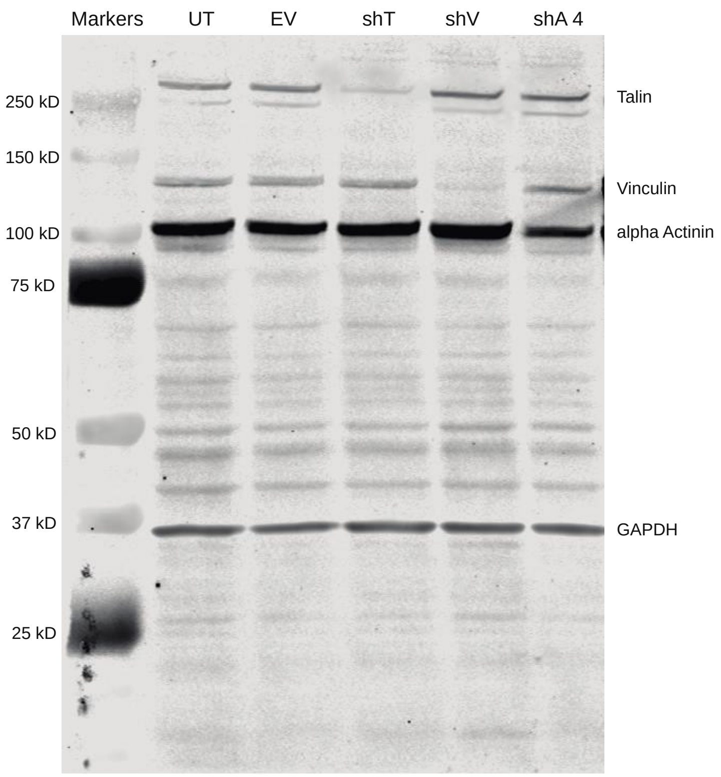

Supplementary Figure 2. The entire immunoblot used to generate Figure 7A. Lysates from Jurkat T cells untransduced or stably expressing the indicated lentiviral constructs were separated by SDS-PAGE and probed by immunoblotting with the indicated antibodies, confirming successful knockdown of Talin, Vinculin and alpha-Actinin 4. UT - untransduced, EV - empty vector, shT - shRNA to Talin, shV - shRNA to Vinculin, shA 4 - shRNA to alpha-Actinin 4.

Supplementary Video Legends

Video S1. Jurkat T cells expressing GFP-actin were imaged by spinning disk confocal microscopy while spreading on glass coverslips coated with anti-CD3 alone. Rendered stacks of three images planes are played back at 20X real time. Scale bar = 10μm

Video S2. Jurkat T cells expressing GFP-actin were imaged by spinning disk confocal microscopy while spreading on glass coverslips coated with anti-CD3 + VCAM-1. Rendered stacks of three images planes are played back at 20X real time. Scale bar = 10μm

Video S3. Jurkat T cells expressing GFP-actin were imaged by spinning disk confocal microscopy while spreading on glass coverslips coated with anti-CD3 + ICAM-1. Rendered stacks of three images planes are played back at 20X real time. Scale bar = 10μm

Video S4. Primary human CD4+ T cells expressing GFP-Lifeact were imaged by spinning disk confocal microscopy while spreading on glass coverslips coated with anti-CD3 alone. Rendered stacks of three images planes are played back at 20X real time. Scale bar = 10μm

Video S5. Primary human CD4+ T cells expressing GFP-Lifeact were imaged by spinning disk confocal microscopy while spreading on glass coverslips coated with anti-CD3 + VCAM-1. Rendered stacks of three images planes are played back at 20X real time. Scale bar = 10μm

Video S6. Primary human CD4+ T cells expressing GFP-Lifeact were imaged by spinning disk confocal microscopy while spreading on glass coverslips coated with anti-CD3 + ICAM-1. Rendered stacks of three images planes are played back at 20X real time. Scale bar = 10μm

Video S7. Primary human CD4+ T cells expressing GFP-Lifeact were imaged by spinning disk confocal microscopy while spreading on glass coverslips coated with anti-CD3 + ICAM-1 + VCAM-1. Rendered stacks of three images planes are played back at 20X real time. Scale bar = 10μm

Video S8. Jurkat T cells expressing GFP-actin and an empty shRNA control vector were imaged by spinning disk confocal microscopy while spreading on glass coverslips coated with anti-CD3 alone. Rendered stacks of three images planes are played back at 20X real time. Scale bar = 10μm

Video S9. Jurkat T cells expressing GFP-actin and an empty shRNA control vector were imaged by spinning disk confocal microscopy while spreading on glass coverslips coated with anti-CD3 + VCAM-1. Rendered stacks of three images planes are played back at 20X real time. Scale bar = 10μm

Video S10. Jurkat T cells expressing GFP-actin and suppressed for talin were imaged by spinning disk confocal microscopy while spreading on glass coverslips coated with anti-CD3 + VCAM-1. Rendered stacks of three images planes are played back at 20X real time. Scale bar = 10μm

Video S11. Jurkat T cells expressing GFP-actin and suppressed for vinculin were imaged by spinning disk confocal microscopy while spreading on glass coverslips coated with anti-CD3 + VCAM-1. Rendered stacks of three images planes are played back at 20X real time. Scale bar = 10μm
